# Structural basis of βTrCP1-associated GLI3 processing

**DOI:** 10.1038/s41598-019-43392-3

**Published:** 2019-05-03

**Authors:** Shagufta Shafique, Sajid Rashid

**Affiliations:** National Center for Bioinformatics, Quaid I Azam University, Islamabad, Pakistan

**Keywords:** Protein structure predictions, Ubiquitylation

## Abstract

Controlled ubiquitin-mediated protein degradation is essential for various cellular processes. GLI family regulates the transcriptional events of the sonic hedgehog pathway genes that are implicated in almost one fourth of human tumors. GLI3 phosphorylation by Ser/Thr kinases is a primary factor for their transcriptional activity that incurs the formation of both GLI3 repressor and activator forms. GLI3 processing is triggered in an ubiquitin-dependent manner via SCF^βTrCP1^ complex; however, structural characterization, mode of action based on sequence of phosphorylation signatures and induced conformational readjustments remain elusive. Here, through structural analysis and molecular dynamics simulation assays, we explored comparative binding pattern of GLI3 phosphopeptides against βTrCP1. A comprehensive and thorough analysis demarcated GLI3 presence in the binding cleft shared by inter-bladed binding grooves of β-propeller. Our results revealed the involvement of all seven WD40 repeats of βTrCP1 in GLI3 interaction. Conversely, GLI3 phosphorylation pattern at primary protein kinase A (PKA) sites and secondary casein kinase 1 (CK1) or glycogen synthase kinase 3 (GSK3) sites was carefully evaluated. Our results indicated that GLI3 processing depends on the 19 phosphorylation sites (849, 852, 855, 856, 860, 861, 864, 865, 868, 872, 873, 876, 877, 880, 899, 903, 906, 907 and 910 positions) by a cascade of PKA, GSK3β and CSKI kinases. The presence of a sequential phosphorylation in the binding induction of GLI3 and βTrCP1 may be a hallmark to authenticate GLI3 processing. We speculate that mechanistic information of the individual residual contributions through structure-guided approaches may be pivotal for the rational design of specific and more potent inhibitors against activated GLI3 with a special emphasis on the anticancer activity.

## Introduction

Protein modification by ubiquitination plays a central role in multiple cellular processes including signal transduction, cell cycle progression, and metabolic pathways^1^. The addition of ubiquitin to numerous fundamental components is involved in the regulation of Hedgehog (Hh) signaling pathway^2^. Hh signaling encompasses a wide range of cellular and molecular mechanisms, such as protein trafficking, protein-protein interactions and post-translational modifications including phosphorylation, lipidation and proteolytic cleavage^3^. The family of secreted Hh signaling molecules plays essential roles in the morphogenesis, homeostasis, cell growth and patterning of numerous embryonic structures of animals ranging from flies to humans^4,5^. It also controls morphogenesis and homeostasis of various tissues comprising vertebrate and invertebrate epidermal appendages^5,6^. In *Drosophila*, Hh signaling is stimulated through a transcription factor Cubitus interruptus (Ci), known to have a central zinc-finger DNA-binding domain^4,7^. In vertebrates, the function of Ci has been expanded to three GLI proteins: GLI1, GLI2 and GLI3^8^. GLI proteins activate the transcription of several target genes that are involved in numerous aspects of tumorigenesis^9,10^. GLI3 primarily coordinates Hh signaling by functioning as activator or repressor depending upon the presence or absence of Hh^11^. GLI homologues exhibit distinctive but overlapping roles. GLI1 works only as a transcriptional activator, whereas GLI2 and GLI3 can act as activators as well as repressors of Hh target genes^4,12^.

Any imbalance of Hh pathway activity including the key transcription effector GLI has been implicated in many human disorders including cancer^9,11^. The constitutive activation of the Hh signaling pathway is a potential mediator of colon, glioma, medulloblastoma, basal cell carcinoma, lung cancer, esophageal cancer, gastric cancer, pancreatic cancer, breast cancer and tumors^10,12–16^. GLI activity is controlled by diverse regulatory processes; among them most prominent is the ubiquitin-mediated proteolysis. Ubiquitin modification of the GLI transcription factors is a vital mechanism to suppress Hh pathway activity. In the absence of Hh, limited degradation of GLI3 converts it into carboxyl-terminal truncated form that functions as a transcriptional repressor of Hh pathway^17^.

**Figure 1:**
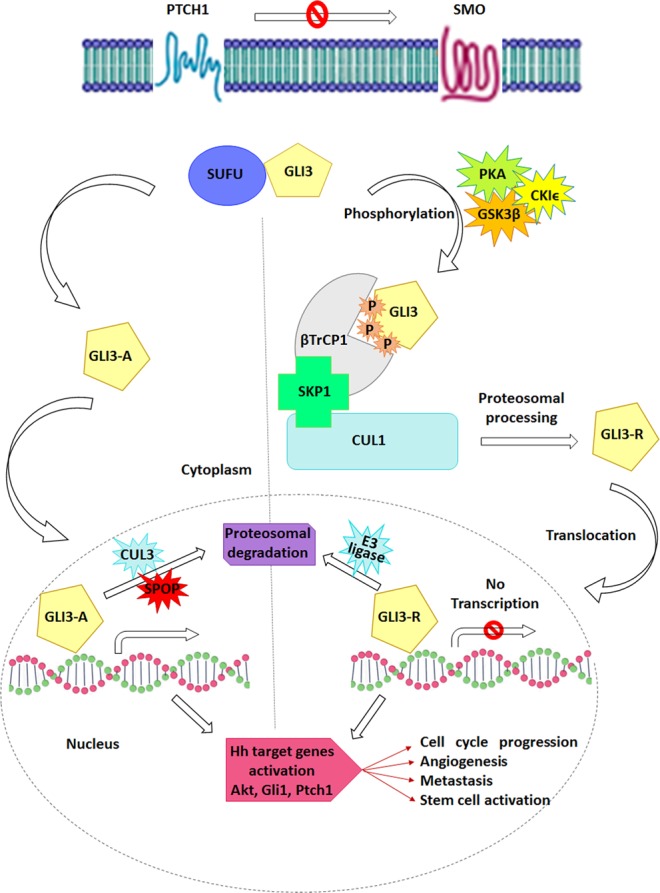
Schematic illustration of GLI3 translocation, processing, and degradation via SCF (SKP1, Cullin, F-box protein complex). In the absence of hedgehog (right panel), Ptch1 constitutively inhibits Smo, preventing its ciliary localization. In this state, GLI proteins are retained in a complex with Sufu, where PKA, CK1ε, and GSK3 phosphorylate them. Phosphorylated GLI3 binds the SCF complex that is partially ubiquitinated and processed by the proteasome into GLI3-R, which translocates to the nucleus and represses transcription of Hh target genes, including Akt, Gli1, Ptch1 etc., prior to degradation by an unknown E3 ligase complex. In the presence of Hh (Left panel), PKA phosphorylation is inhibited. The activated form of GLI3 not only prevents it from processing but also permits its subsequent transport to the nucleus to allow activation of transcription of Hh target genes. Following this, GLI3-A is ubiquitinated by the SPOP/CUL3 complex and degraded by the proteasome. P, phosphate group; Ptch1, Patched 1; SUFU, Suppressor of Fused; SMO, Smoothened; PKA, Protein kinase A, GSK3, Glycogen synthase kinase 3; CSKI, Casein kinase 1; CUL1, Cullin 1; CUL3, Cullin 3; RBX1, RING-box protein 1; SKP1, S-Phase Kinase-Associated Protein 1; SPOP, Speckle Type BTB/POZ Protein; GLI3-R, GLI3 repressor; GLI3-A, GLI3 activator.

GL3 processing entails its extensive phosphorylation by three Ser/Thr kinases i.e. protein kinase A (PKA), glycogen synthase kinase 3 (GSK3), and casein kinase I (CKI) and component of SCF (SKP1, Cullin, F-box containing complex) E3 ligase i.e. βTrCP1, CUL1 (Cullin1) and RBX1 (RING-box protein 1)^7,8,18^. In the absence of Hh signaling, GLI3 processing is provoked by PKA-dependent phosphorylation, which is prerequisite for the later phosphorylation events by GSK3 and CK1 leading to direct binding and ubiquitination by SCF^βTrCP1^ ^16,17^. In the presence of Hh signaling, GLI3 is released from PKA/GSK3-mediated phosphorylation to activate Hh target genes^1,17,18^ as shown in Fig. 1. Recently, it has been reported that GLI3 processing is mediated by sequential phosphorylation of βTrCP1-binding sites through PKA, GSK3β and CK1 enzymes^16,17^. GLI3 central domain comprises four PKA sites; the first PKA site is flanked by a CK1 site, while second, third and fourth sites are flanked by both GSK3 and CK1 sites^8^. GSK3β and CK1 trigger multiphosphorylation of GLI3, where GSK3β phosphorylates Ser 4 residue N-terminal to a phosphoserine, while CK1 phosphorylates Ser 3 residue C-terminal to a phosphoserine^17^. βTrCP1 explicitly binds to its substrates via the phosphodegron motif i.e. DSpGX_2–4_Sp, where Sp denotes phosphorylated serine and X denotes any residue^19^. βTrCP1 binding recruits the ubiquitination machinery to its substrates ensuing either degradation or processing. GLI3 binding necessitates 4 βTrCP1-binding sites (SSASTIS, SSAYLS, SSGISPCFS and DSYDPIS; referred as motifs 1–4) that are related to DSGX2–4S motif lying in the substrates of βTrCP1^17^. GLI3 processing depends on the phosphorylation of 4 PKA sites (Ser849, Ser865, Ser877 and Ser907), further phosphorylated by CK1 (Ser852, Ser868, Ser880 and Ser910) and GSK3β (Ser861, Ser873 and Ser903). CK1-phosphorylation at Ser855 (motif-1) primes further phosphorylations by GSK3β at secondary sites including Ser856 and Ser860 (motif-2), Ser872 (motif-3) and Ser899 (motif-4) residues in addition to phosphorylation at Ser864, Ser876 and Ser906 residues^17^.

Despite the contemporary range of βTrCP1 substrates, molecular significance of βTrCP1 binding to the multiple motifs in GLI3 is not clearly understood. Here in current study, we utilized *in silico* approaches to elucidate the structural basis of GLI3 phosphorylation in the regulation of βTrCP-mediated protein degradation. Unlike other substrates of E3 ligases, GLI3 requires PKA, GSK3 and CKI accessory proteins for phosphorylation and ubiquitination by βTrCP1. Our detailed structural analyses highlighted significant conformational switches and noteworthy residual contributions upon phosphorylation and binding. Thus by exploring the association of βTrCP1 and GLI3, our study may provide invaluable insight in understanding the Hh relationship with cancer pathogenesis.

## Methodology

### Data set

Primary protein sequence of GLI3 (UniProt ID: P10071) was retrieved through UniProtKB/Swiss-Prot database (http://www.uniprot.org/). Three dimensional (3D) coordinates of human βTrCP1 (PDB ID: 1P22; 2.95 Å resolution) were retrieved through Protein Data Bank^20^. 3D-structure of GLI3 central domain (846–910AA) was predicted by PEP-FOLD^21^, which is a *de novo* approach, used for the prediction of small peptide structures. MolProbity tool^22^ was utilized to validate 3D model followed by model refinement using WinCoot^23^. Structure editing (phosphorylation) was carried out using UCSF Chimera 1.11.2^24^.

Phosphopeptides were generated by adding phosphate groups to serine residues lying at the degradation motif through UCSF Chimera 1.11.2. In GLI3^PKA^ phosphopeptide, phosphorylation was performed at Ser849, Ser865, Ser877 and Ser907, GLI3^GSK3β^ phosphopeptide exhibited phosphorylation at Ser861, Ser873 and Ser903 residues, while in GLI3^CKIϵ^ phosphopeptide, Ser852, Ser868, Ser880 and Ser910 residues were phosphorylated. In addition to these residues, GLI3-β1 peptide exhibited phosphorylation at Ser855 residue, GLI3-β2 peptide was generated by the addition of phosphate groups at Ser856, Ser860 and Ser864 residues, GLI3-β3 peptide contained phosphate groups at Ser872 and Ser876 residues, GLI3-β4 peptide exhibited phosphate groups at Ser899 and Ser906 residues, while GLI3-β1–4 was generated by adding phosphate groups at Ser855, Ser856, Ser860, Ser864, Ser872, Ser876, Ser899 and Ser906 residues. Structures were optimized by energy minimization through UCSF Chimera 1.11.2 and GROMACS^25^ using Amber force field. Energy minimization was performed using the steepest descent algorithm for 50000 steps (with a lower dt value of 0.001). Ideally, the maximum force *Fmax* (gradient of the potential) should be less than 1000 kJ mol^−1^ nm^−2^. In our case, *Fmax* <1000 was achieved at 543 and 687 steps with potential energy values of −1.07452e + 06 and −1.284026e + 06 for βTrCP1 in complex with GLI3-β1–4 and GLI3-β4 peptides, respectively. All systems were converged at same range.

### Molecular docking analysis

In order to explore the binding pattern of βTrCP1 and GLI3 peptides (GLI3-un, GLI3-β1, GLI3-β2, GLI3-β3, GLI3-β4 and GLI3-β1–4), molecular docking was accomplished by HADDOCK^26^. Prior to docking analysis, 3D structures of βTrCP1 and GLI3 peptides were submitted to CPORT server to predict the interface residues^27^. CPORT provides a list of active and passive residues of the interaction interfaces that are further employed for the preparation of ambiguous interaction restraints (AIR) by HADDOCK^28,29^. Docking simulations were carried out with default parameters, among them HIS protonation state, random removal of restraints, number of structures to dock and refine, electrostatic scaling constant, restraint energy constants, scoring parameters, temperature and time steps of refinement processes were defined automatically by the interface of HADDOCK server^30^. HADDOCK produces 1000 docked structures through experimental data to drive docking. HADDOCK scores each model using Equation 1 and retains the top 200 solutions for consequent flexible refinement, where E_AIR_, E_elec_, E_vdW_ and E_desolv_ are the restraints violation , electrostatic, van der Waals and desolvation energies, respectively. BSA is the buried surface area and E_data_ indicates the energy of other restricted data. HADDOCK score is weighted as the sum of the following four terms: electrostatic energy (weight: 0.2), Van der Waals energy (weight: 1.0), desolvation energy (weight: 1.0) and restraint violation energy (weight: 0.1).

1 $${\rm{E}}=0.01\,{{\rm{E}}}_{{\rm{vdW}}}+0.1\,{{\rm{E}}}_{{\rm{elec}}}+0.01\,{{\rm{E}}}_{{\rm{AIR}}}\,-\,0.01\,{\rm{BSA}}+1.0\,{{\rm{E}}}_{{\rm{desolv}}}+0.1\,{{\rm{E}}}_{{\rm{data}}}$$

Furthermore, selected models are subjected to a semiflexible refinement followed by water refinement step in torsion angle space and explicit water shell, respectively. These parameters are scored using Equations 2 and 3, respectively. The solutions are clustered using a 7.5 Å limit created on their pairwise ligand interface. Root mean square deviation (RMSD) values and cluster ranks are rendered to the average score of the top four structures in each cluster.

2 $${\rm{E}}=1.0\,{{\rm{E}}}_{{\rm{vdW}}}+1.0\,{{\rm{E}}}_{{\rm{elec}}}+0.1\,{{\rm{E}}}_{{\rm{AIR}}}\,-\,0.01\,{\rm{BSA}}+1.0\,{{\rm{E}}}_{{\rm{desolv}}}+0.1\,{{\rm{E}}}_{{\rm{data}}}$$

3 $${\rm{E}}=1.0\,{{\rm{E}}}_{{\rm{vdW}}}+0.2\,{{\rm{E}}}_{{\rm{elec}}}+0.1\,{{\rm{E}}}_{{\rm{AIR}}}+1.0\,{{\rm{E}}}_{{\rm{desolv}}}+0.1\,{{\rm{E}}}_{{\rm{data}}}$$

The best docked complexes of top ranked clusters were selected and visualized by UCSF Chimera 1.11.2 to analyze their conformational readjustments. Residual interactions such as hydrogen bonds, hydrophobic, electrostatic interactions and bond lengths were evaluated by employing DIMplot embedded in LigPlus^31^. Furthermore, these complexes were subjected to molecular dynamic simulations for detailed analysis.

### Molecular dynamics simulation analysis

In order to gain a deep insight into the mechanism of GLI3 phosphodegron recognition by apo-βTrCP1 and its bound forms with GLI3-β1, GLI3-β2, GLI3-β3, GLI3-β4 and GLI3-β1–4, these complexes were subjected to molecular dynamics (MD) simulation assays to evaluate the stability, folding, conformational changes and dynamic behavior of interacting proteins. Groningen Machine for Chemicals Simulations (GROMACS) 5.1.4 package was used to perform MD simulation assay. All MD simulations were performed by GROMOS96 43a1 force field^32^ to acquire the equilibrated system. All systems were solvated using SPC water model^33^ in a periodic box, followed by the addition of Na^+^ and Cl^−^ counter ions to neutralize the system. Before MD simulations, systems were subjected to energy minimization to remove initial steric clashes using 1000 steps of steepest descent algorithm via a tolerance of 10 KJ/mol/nm^−1^. Systems were equilibrated for 1000 ps at 300 K and 1 bar pressure in NVT^34^ and NPT^35^ ensembles, respectively and their equilibration profiles were evaluated (Fig. S1). The hydrogen bond length constraints were employed at a time step of 2 fs for numerical integration with Verlet leap-frog algorithm^36^. PME (Particle Mesh Ewald) algorithm^37^ was employed to evaluate the long-range (LR) electrostatic interactions. Finally, MD simulations were run for 40 ns time scale. Stability and time-dependent behavior of each system were investigated using system conformations extracted every 5 ns from the MD trajectories and analyzed using UCSF Chimera and GROMACS tools. GROMACS modules such as *g_rms*, *g_rmsf*, *g_energy* and *g_hbond* were utilized to analyze the stability and behavior of each system. The secondary structure database (DSSP) was utilized to analyze the time-dependent secondary structure fluctuations in the bound complexes^38^.

### Binding free energy calculation

Poisson-Boltzmann or generalized Born and surface area continuum solvation (MM/PBSA) method^39^ was employed to calculate the binding free energy of the system. This method provides inclusive energy composition and improves docking energy via incorporating protein flexibility. The binding energy of ligand-protein complex was calculated using the following equation:

4 $$\Delta {G}_{binding}={G}_{complex}-({G}_{protein}+{G}_{ligand})$$

*G*_*complex*_ is the total free energy of the protein-ligand complex; *G*_*protein*_ and *G*_*ligand*_ are total energy of separated protein and ligand in solvent, respectively. The free energy for each individual *G*_*complex*,_ *G*_*protein*_ and *G*_*ligand*_ were estimated by:

5 $${G}_{x}=({E}_{MM}+{G}_{solvation})$$

*x* is the protein-ligand complex. *E*_*MM*_ is the molecular mechanics energy and *G*_*solvation*_ is free energy of solvation. The molecular mechanics potential energy was calculated in vacuum as following:

6 $${E}_{MM}={E}_{bonded}+{E}_{non-bonded}={E}_{bonded}+({E}_{vdw}+{E}_{elec})$$

*E*_*bonded*_ is bonded interaction including bond, angle, dihedral and improper interactions and *E*_*non-bonded*_ is non-bonded interactions that consist of van der Waals (*E*_*vdw*_) and electrostatic (*E*_*elec*_) interactions.The solvation free energy (*G*_*solvation*_) was estimated as the sum of electrostatic solvation free energy (*G*_*polar*_) and apolar solvation free energy (*G*_*non-polar*_):

7 $${G}_{solvation}={G}_{polar}+{G}_{non-polar}$$

*G*_*polar*_ was computed using the Poisson-Boltzmann (PB) equation^40^ and G_*non-polar*_ was calculated using a solvent accessible surface area (SASA) as follows:

8 $${G}_{non-polar}={\rm{\gamma }}SASA+b$$

γ is a coefficient related to surface tension of the solvent and b is fitting parameter.

## Results

### Structural evaluation of GLI3 peptides

The predicted structures of GLI3 peptides (GLI3-β1, GLI3-β2, GLI3-β3, GLI3-β4 and GLI3-β1–4) were evaluated by Ramachandran plots (Fig. S2), where blue colour indicated favorable region (sterically allowed regions), while no outliers were observed. Approximately, 92–95% residues were resided in the blue region. Additionally, parameters like peptide bond planarity, non-bonded interactions, Cα-tetrahedral distortion, main chain H-bond energy values and overall G-factors for the predicted models were lying in the favourable ranges. GLI3 peptide structures optimized through GROMACS tool were further evaluated by RAMPAGE^41^.

### Phosphopeptide binding and conformational transitions

In order to evaluate mechanism of substrate recognition by βTrCP1, GLI3 phosphopeptides were subjected to molecular docking analysis. Given a maximum number of 200 models for clustering, HADDOCK clustered 99 structures of βTrCP1-GLI3-un complex in 15 clusters, 86 structures of βTrCP1-GLI3^PKA^ complex in 8 clusters, 66 structures of βTrCP1-GLI3^GSK3β^ complex in 11 clusters, 72 structures of βTrCP1-GLI3^CKIϵ^ complex in 11 clusters, 130 structures of βTrCP1-GLI3-β1 complex in 16 clusters, 115 structures of βTrCP1- GLI3-β2 complex in 15 clusters, 93 structures of βTrCP1- GLI3-β3 complex in 11 clusters, 117 structures of βTrCP1- GLI3-β4 complex in 15 clusters and 114 structures of βTrCP1-β1–4 complex in 14 clusters, representing 49.5%, 43.0%, 33.0%, 36.0%, 65.0%, 57.5%, 46.5%, 58.5% and 57.0% of water-refined models, respectively. The statistics of top 10 clusters (ranked on the basis of lowest overall energy and Z-score values) were shown by HADDOCK, out of which scores of the optimal clusters for each βTrCP1-peptide complexes are illustrated in Table 1. The more negative HADDOCK and Z-scores indicate a reliable interaction. Z-score is the quantitative measure of cluster standard from the average score.

**Table 1:** HADDOCK scoring functions of optimal clusters.

	Parameters						
	HADDOCK score	Z-score	Van der Waals energy (kcal/mol)	Electrostatic energy (kcal/mol)	Desolvation energy (kcal/mol)	Restraints violation energy (kcal/mol)	Buried Surface Area Å^2^
GLI3_un	−22.6 +/− 11.8	−1.7	−91.8 +/− 4.6	−536.0 +/− 36.4	24.1 +/− 6.6	1522.7 +/− 154.21	2977.2 +/− 93.1
GLI3^PKA^	−17.6 +/− 16.1	−1.9	−71.4 +/− 2.8	−520.5 +/− 18.2	21.2 +/− 4.0	1366.4 +/− 197.36	2447.2 +/− 156.8
GLI3^GSK3β^	−2.2 +/− 10.3	−1.4	−90.9 +/− 8.3	−340.8 +/− 37.2	14.9 +/− 5.5	1419.3 +/− 98.96	2523.1 +/− 75.0
GLI3^CSK1ε^	3.2 +/− 17.3	−2.3	−85.5 +/− 6.5	−310.1 +/− 33.1	7.2 +/− 4.9	1435.0 +/− 100.13	2405.9 +/− 94.1
GLI3-β1	−55.3 +/− 17.3	−1.9	−78.6 +/− 7.2	−725.2 +/− 66.6	21.4 +/− 12.1	1470.0 +/− 81.60	2748.0 +/− 184.0
GLI3-β2	−38.2 +/− 21.0	−1.6	−52.7 +/− 6.4	−737.4 +/− 59.4	24.7 +/− 3.3	1372.3 +/− 96.36	2408.2 +/− 134.3
GLI3-β3	−37.3 +/− 9.1	−1.6	−66.8 +/− 6.9	−726.5 +/− 103.6	20.1 +/− 6.1	1545.8 +/− 221.39	2658.3 +/− 79.2
GLI3-β4	−91.6 +/− 9.9	−2.4	−75.6 +/− 19.2	−940.2 +/− 88.3	30.8 +/− 9.0	1412.2 +/− 211.22	2723.6 +/− 260.0
GLI3-β1–4	−65.6 +/− 12.4	−2.2	−64.9 +/− 6.7	−967.0 +/− 43.3	40.1 +/− 8.5	1526.3 +/− 162.52	2607.7 +/− 104.5

**Figure 2:**
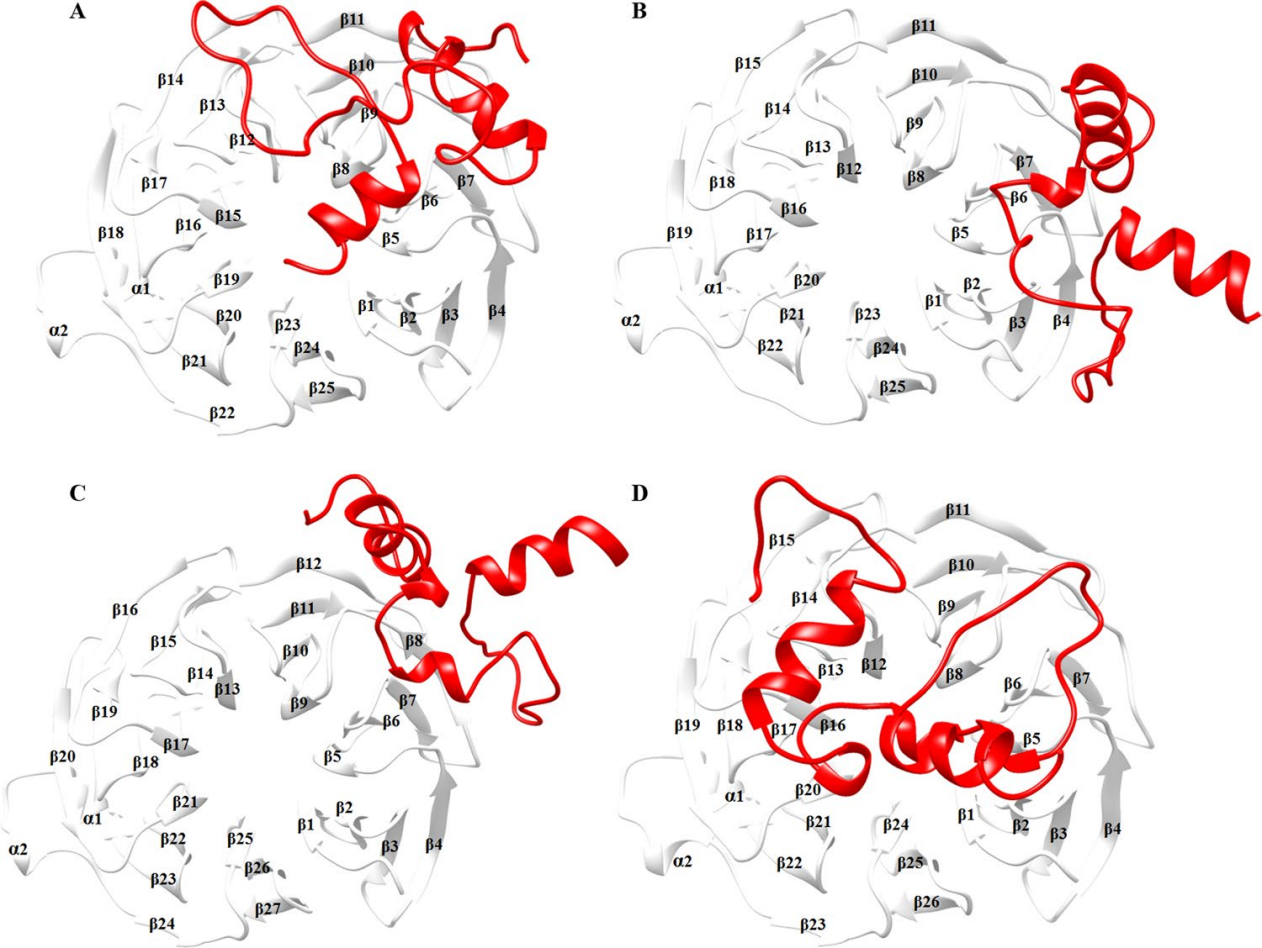
Binding orientation of β-propeller due to phosphopeptide binding. 7 WD40 repeats of βTrCP1, comprising 25 beta sheets are organized to form a circular structure (β-propeller). Optimal docked complexes of βTrCP1 bound (**A**) GLI3^PKA^ (**B**) GLI3^GSK3β^, (**C**) GLI3^CSKIϵ^ (**D**) GLI3-β1–4. βTrCP1 is shown in white colored ribbon, while GLI3 phosphopeptide is shown in red colored ribbon.

All βTrCP1-peptide complexes were carefully characterized to access their binding patterns. In case of βTrCP1-GLI3^PKA^ complex, phosphopeptide exhibited binding with the 1st, 2nd and 7th WD40 repeats of βTrCP1 having a score of −17.6 (Fig. 2A). In contrast, GLI3^GSK3β^ and GLI3^CKIϵ^ peptides did not exhibit binding with βTrCP1 (Fig. 2B,C). In βTrCP1 and GLI3-β1–4 complex, phosphopeptide binding was observed at the upper interface of β-propeller (Fig. 2D). Thus GLI3 phosphorylation by all three enzymes (PKA, GSK3β and CKIε) resulted in accurate binding with βTrCP1 substrate binding site.

**Figure 3:**
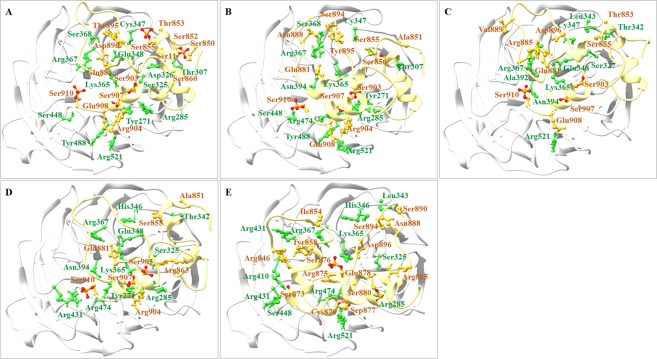
Binding mode and molecular interaction analysis of motif peptides. Optimal docked complexes of βTrCP1-bound (**A**) GLI3-β1 (**B**) GLI3-β2, (**C**) GLI3-β3 (**D**) GLI3-β4 and (**E**) GLI3-β1–4 peptides. βTrCP1 and GLI3 are shown in white and khaki colored ribbons with interacting residues in green and goldenrod colored ball and sticks, respectively.

Next, we examined GLI3-β1, GLI3-β2, GLI3-β3, GLI3-β4 and GLI3-β1–4-bound βTrCP1 complexes to explore the intricate details of phosphopeptide binding. Evidently, docking clusters of phosphopeptides at the substrate binding pocket of βTrCP1 revealed predominant binding affinities for WD40 repeats (Fig. 3). The individual residues involved in interactions were evaluated through DIMPLOT and UCSF Chimera 1.11.2. These residual contributions specified that almost all 7 WD40 repeats imparted equal propensity to bind with GLI3 phosphopeptides. Though, it is vague at the moment whether binding of peptide results in any notable modification in the βTrCP1functioning. The binding residues as listed in Table 2. In comparison to other complexes, βTrCP1-GLI3-β1–4 complex exhibited more number of hydrogen bonds. Phosphorylated residues (Sep873, Sep876, Sep877 and Sep880) of GLI3 contributed in interaction with all 7 WD40 repeats of βTrCP1. As reported by Wu *et al*., 2003^42^, βTrCP1-specific residues (Tyr271, Arg285, Ser309, Leu311, Ser325, Leu351, Asn394, Arg431, Gly432, Ala434, Ser448, Leu472, Arg474, Tyr488 and Arg521) involved in phosphorylated β-catenin peptide binding were consistent in GLI3-βTrCP1 complex, where GLI3-β1–4 peptide binding was evident at the upper face of β-propeller (Fig. 3). These results indicate that βTrCP1 shares common region upon interaction with phosphor-substrates.

**Table 2:** Binding residues of βTrCP1 and GLI3 phosphopeptides. Residues involved in hydrogen bonding and hydrophobic associations are indicated in bold and normal forms, respectively.

Complex	βTrCP1 residues	GLI3 residues
βTrCP1 and GLI3-β1	**Tyr271**, **Arg285**, **Gly305**, **Arg307**, **Ser325**, **Asp326**, Ser327, Arg330, Met339, **Leu343**, Ile344, His345, His346, **Cys347**, **Glu348**, **Lys365**, Asp366, **Arg367**, **Ser368**, Val385, Val387, **Ser448**, **Tyr488**, **Arg521**	**Ser850**, Ala851, **Sep852**, **Thr853**, Ile854, **Sep855**, **Ser856**, **Ser860**, **Gln881**, Val889, Ser890, Val891, Ala892, Ser894, **Tyr895**, **Asp896**, Ile898, **Sep903**, **Arg904**, **Sep907**, **Glu908**, **Sep910**
βTrCP1 and GLI3-β2	**Tyr271**, **Arg285**, **Thr307**, **Ser327**, Arg330, Ile344, **Cys347**, Glu348, **Lys365**, Asp366, **Arg367**, **Ser368**, Val387, Gly388, His389, Arg390, **Asn394**, **Gly432**, **Ser448**, **Arg474**, **Tyr488**, **Arg521**	Sep849, **Ala851**, Thr853, **Ser855**, **Sep856**, **Gln881**, **Asn888**, Val889, Val891, Ala892, **Ser894**, **Tyr895**, Pro897, **Sep903**, **Arg904**, **Sep907**, **Glu908**, **Sep910**
βTrCP1 and GLI3-β3	Thr307, **Ser327**, Thr328, Arg330, Met339, **Thr342**, **Leu343**, Ile344, His345, **Cys347**, **Glu348**, **Lys365**, **Arg367**, Ser368, Val385, Val387, **Gly388**, **Ala392**, **Asn394**, **Arg521**	Ala851, Sep852, **Thr853**, Ile854, **Ser855**, Ser856, **Gln881**, **Arg885**, **Val889**, Ser890, Val891, Ala892, Asp893, Tyr895, **Asp896**, Ile898, **Sep903**, Ser906, **Sep907**, **Glu908**, **Sep910**
βTrCP1 and GLI3-β4	**Tyr271**, **Arg285**, Thr307, **Ser325**, Asp326, **Thr342**, Ile344, **His346**, Cys347, **Glu348**, **Lys365**, **Arg367**, Val387, **Asn394**, **Arg431**, **Ser448**, **Arg474**, Tyr488	**Ala851**, Sep852, **Ser855**, Leu859, Ser860, **Arg863**, **Gln881**, Ala892, Tyr895, Ile898, **Sep903**, **Arg904**, **Sep907**, Glu908, **Sep910**
βTrCP1 and GLI3-β1-4	**Arg285**, Thr307, Gly308, **Ser325**, Ser327, **Leu343**, Ile344, **His346**, **Cys347**, **Lys365**, **Arg367**, **Arg390**, Asn394, **Arg410**, **Arg431**, **Gly432**, **Ala434**, **Ser448**, **Arg474**, **Arg521**	**Arg847**, **Ile854**, **Tyr858**, **Cys870**, **Sep873**, **Arg875**, **Sep876**, **Sep877**, **Glu878**, Ala879, **Sep880**, **Arg885**, Pro886, **Asn888**, Val889, **Ser890**, Val891, Asp893, **Ser894**, **Asp896**
βTrCP1 and GLI3^PKA^	**Arg285**, **Ser309**, Leu311, Ser325, **Asp326**, **Ser327**, Leu343, **Ile344**, **His345**, His346, **Cys347**, **Glu348**, Ala349, **Lys365**, Asp366, **Arg367**, Ser368, Trp372, Ile380, Thr381, Leu382, Val387, **Gly388**, His389, **Arg390**, **Tyr488**, **Arg521**	**Sep849**, Ser850, **Ala851**, Ser852, Thr853, **Ser855**, Ser856, Leu859, Ser860, **Arg863**, **Gln881**, **Ser890**, Ala892, Asp893, **Ser894**, **Tyr895**, **Asp896**, Pro897, Ile898, **Ser903**, **Arg904**, **Sep907**, **Glu908**, **Ala909**, **Ser910**
βTrCP1 and GLI3^GSK3β^	**Glu265**, Thr266, Arg285, **Asn287**, Thr288, **Lys290**, **Cys299**, Ile302, **Thr304**, Gly305, His306, **Thr307**, Gly308, Ser309, **Ser325**, Asp326, **Ser327**, Thr328, Arg330, Trp332, **Met339**, Asn341, **Thr342**, Leu343, Ile344, **His346**, **Cys347**, **Glu348**, **Lys365**	Arg846, **Arg847**, Asp848, **Sep849**, Ser850, Thr853, **Ile854**, Ser855, **Tyr858**, Arg862, **Sep873**, **Arg874**, Arg875, **Sep876**, **Arg885**, Asn888, **Ser890**, Val891, Asp893, **Ser894**, **Tyr895**, **Asp896**, Pro897
βTrCP1 and GLI3^CSKIϵ^	**Thr307**, Gly308, **Asp326**, **Thr328**, **Arg330**, Glu338, Met339, Leu340, **Asn341**, **Thr342**, Leu343, Ile344, **His345**, **His346**, **Cys347**, **Asp366**, **Ser368**, Trp372, **Thr378**, Asp379, **Ile380**, Thr381, **Leu382**, Arg384, Val385, Val387	Arg846, **Arg847**, Asp848, **Thr853**, Ile854, **Ala857**, **Tyr858**, Leu859, **Ser861**, Arg862, **Sep873**, **Arg874**, **Arg875**, **Sep876**, Gln887, Asn888, Ser890, Val891, **Asp893**, Ser894, Tyr895, **Asp896**, Pro897

### Molecular dynamics simulation analysis

**Figure 4:**
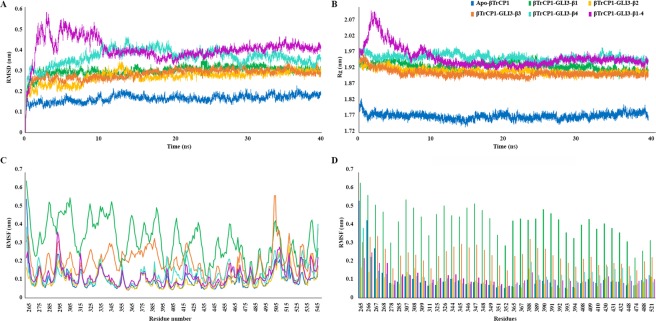
Time-dependent analysis of 40 ns MD simulations of apo- versus GLI3 peptide-bound βTrCP1. (**A**) RMSD plotted as a time function computed through least square fitting of backbone Cα-atoms. (**B**) Rg plots of individual simulated complexes along the course of 40 ns of MD simulation. (**C**) RMSF per residue plot for each trajectory file. (**D**) Comparison of the most fluctuating residues is indicated by bar chart. Apo and bound forms of βTrCP1 with GLI3-β1, GLI3-β2, GLI3-β3, GLI3-β4 and GLI3-β1–4 are represented in blue, green, gold, orange, cyan and purple colors, respectively.

In order to permit elucidation of conformational transitions, dynamic behavior and stability of contacts, complexes of βTrCP1 and phosphorylated peptides (GLI3-β1, GLI3-β2, GLI3-β3, GLI3-β4 and GLI3-β1–4) were further characterized by 40 ns molecular dynamics (MD) simulations. The stability of secondary structure elements and conformational changes of simulated complexes were assessed by plotting RMSD (Root Mean Square Deviation), RMSF (Root Mean Square Fluctuation), hydrogen bonding and binding energy plots. RMSD for each complex was measured throughout 40 ns time scale using apo-form as a reference. Overall RMSD analysis revealed stable behavior for all systems in a range of 2.0–4.2 Å (Fig. 4A). Dynamically, βTrCP1 bound GLI3-β1–4 complex displayed slight increase in deviations during the initial 10 ns time period, compared to other complexes (Fig. 4A). However, later on, backbone RMSD profile for GLI3-β1–4 was quite stable (3.5–4 Å). The pronounced changes in RMSD trend indicated variability in the structural rearrangements upon GLI3 phosphorylation. Correspondingly, Rg profiles of individual systems were consistent with their resultant RMSD profiles (Fig. 4B). A higher Rg value implies lower compactness of a system^43–47^. Consequently, βTrCP1-GLI3-β1–4 exhibited minor compactness than apo-form. Thus higher Rg values of complexes than that of apo-βTrCP1 suggested firmness in the synergic conformational adaptation owing to βTrCP1 interaction.

Subsequent RMSF analysis indicated residual fluctuations at the substrate binding cleft of WD40 repeats (Fig. 4C). βTrCP1 upon binding to GLI3-β1 exhibited significantly higher rate (3–5 Å) of fluctuations as compared to apo and other βTrCP1-bound phosphopeptide forms. In case of GLI3-β3 complex, major fluctuations (up to 3 Å) were detected in βTrCP1 residues, while residues involved in GLI3 binding were comparatively stable (Fig. 4D). In βTrCP1-GLI3-β2 complex, more fluctuations (1.6 Å) were observed in Gly308 and Gly388 residues, while βTrCP1 residues involved in binding remained stable during the course of simulation run. Correspondingly, in βTrCP1-GLI3-β4 complex, major fluctuations were detected in Gly388, His389, Ala392-Asn394, Gly408-Arg410, Lys430-Gly432 and Ser448 (1.7 Å) residues located in the immediate vicinity of binding region (Fig. 4D). Interestingly, all fluctuations were observed in the loop regions. In βTrCP1-GLI3-β1–4 complex, significant fluctuation (2 Å) was observed in Lys268 residue, while βTrCP1 binding residues namely, Arg285, Ser325, Leu343, His346, Cys347, Lys365, Arg367, Arg390, Arg410, Arg431, Gly432, Ala434, Ser448, Arg474 and Arg521 remained stable during the course of simulation run (Fig. 4D).

**Figure 5:**
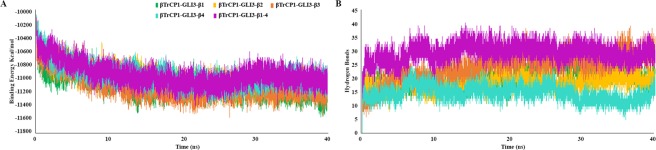
Binding energy and hydrogen bond versus time plots for 40 ns MD simulation. (**A**) LJ-SR binding energy profile. (**B**) Intermolecular hydrogen bonding pattern of βTrCP1-GLI3 complexes. GLI3-β1, GLI3-β2, GLI3-β3, GLI3-β4 and GLI3-β1–4 are represented in green, gold, orange, cyan and purple colors, respectively.

MD simulation trajectory files of βTrCP1-bound phosphopeptide complexes were subjected to energy calculation via LJ-SR (Lennard-Jones Short-Range) binding descriptor. LJ-SR are normal non-bonded interactions within the short-range cutoff. Overall, LJ-SR energy values were quite stable ranging between −10000 to −11500 kcal/mol (Fig. 5A). Analogously, coulomb short range energy values (Coul-SR) are used to assess the system’s equilibration along the simulation run. Coul-SR energy values (−81016 to −87087 kcal/mol) indicated the stability of systems. Furthermore, simulated trajectories of βTrCP1-bound GLI3-β1, GLI3-β2, GLI3-β3, GLI3-β4 and GLI3-β1–4 were examined for hydrogen bond shifts. Inclusively, hydrogen bond interaction pattern remained stable during the entire simulation time (Fig. 5B). The presence of more intermolecular hydrogen bonds in GLI3-β1–4 as compared to other simulated systems indicated enhanced binding of βTrCP1 with GLI3-β1–4 phosphopeptide. Overall, H-bonding pattern inferred stable interactions in agreement with the RMSD distribution (Fig. 4A).

### Conformation change analysis

To monitor the structural changes in apo versus βTrCP1-bound systems, PDB files were extracted every 5 ns (5, 10, 15, 20, 25, 30, 35 and 40 ns) time interval from MD trajectories. During MD simulations, momentous conformational changes were observed at the proximity of central cavity, influencing the peptide binding. The conformational transitions occurring in the β-propellers of βTrCP1 were deeply examined at 30 ns to understand the changes in secondary structural elements (Table 3). Evidently, in GLI3-β1–4-bound βTrCP1, conversion of Thr381-Leu386 β-strand into loop was visible in comparison to other complexes (Table 3). Another change persuaded upon GLI3-β1–4 binding was the extension of 4 β-strands (Arg301-Leu303, Leu313-Tyr315, Ile492-Trp495 and Ile532-Ser534 regions) of βTrCP1 that induced more stability in binding propensity. Moreover, lengths of β12 (Val393-Asp399) and β14 (Phe422-Leu426) strands were reduced; however, these shrinkages did not alter the active site conformation. Another notable secondary structural amendment was witnessed in the loop region of βTrCP1, where Thr540-Trp544 region adopted a β-conformation upon binding to GLI3-β1, GLI3- β2 and GLI3-β1–4 phosphopeptides.

**Table 3:** Secondary structure changes during MD simulations in phosphopeptide-bound βTrCP1 states with reference to apo-βTrCP1.

GLI3-un	GLI3-β1	GLI3-β2	GLI3-β3	GLI3-β4	GLI3-β1–4
Cys272-Tyr275	Val270-Tyr275	Cys272-Tyr275	Val270-Tyr275	Val270-Tyr275	Cys272-Tyr275
Arg301-Leu303	Cys299-Thr304	—	Cys299-Thr304	Arg301-Leu303	Cys299-Thr304
Leu313-Tyr315	—	Leu313-Tyr315	Val310-Tyr315	—	Val310-Gln314
Val319-Gly323	—	Val319-Gly323	Val319-Gly323	Val319-Gly323	Val319-Gly323
Val329-Asp333	—	Val329-Asp333	Val329-Asp333	Val329-Asp333	Val329-Asp333
Met339-Leu343	—	Met339-Leu343	Met339-Leu343	—	Met339-Leu343
Val350-Ser364	Val350-Ser364	Val350-Ser364	His352-Ser364	His352-Cys363	Val350-Ser364
Thr381-Leu386	Thr381-Leu386	Thr381-Leu386	Thr381-Leu386	Thr381-Leu386	—
Val393-Asp399	Val393-Asp399	Val395-Asp399	Val395-Asp397	Val395-Asp399	Val396-Asp399
Phe422-Leu426	Phe422-Leu426	Phe422-Leu426	Phe422-Leu426	Arg424-Leu426	Arg424-Leu426
Cys435-Arg439	Ile433-Arg439	Cys435-Arg439	Cys435-Arg439	Ile433-Tyr438	Cys435-Arg439
Ala461-Leu466	Arg464-Leu466	Val465-Glu467	Ala461-Leu466	Arg464-Glu467	Ala461-Leu466
Val473-Phe478	Val473-Phe478	Ile476-Phe478	Val473-Phe478	Val473-Phe478	Val473-Phe478
Ile492-Trp495	Lys491-Asp496	Lys491-Asp496	Lys491-Asp496	Lys491-Asp496	Lys491-Asp496
Leu497-Leu501	Leu497-Leu501	Leu497-Leu501	—	Leu497-Leu501	Leu497-Leu501
—	Ala507-Leu510	Ala507-Leu510	—	Ala507-Leu510	—
Cys511-Leu515	—	Cys511-Leu515	Cys511-Leu515	Cys511-Leu515	Cys511-Leu515
Leu525-Phe527	Leu525-Phe527	—	Arg524-Asp528	Leu525-Phe527	Leu525-Phe527
Ile532-Ser534	Gln531-Ser535	Ile532-Ser534	Gln531-Ser535	—	Ile532-Ser536
—	Ile541-Trp544	Leu542-Trp544	—	—	Thr540-Trp544

**Figure 6:**
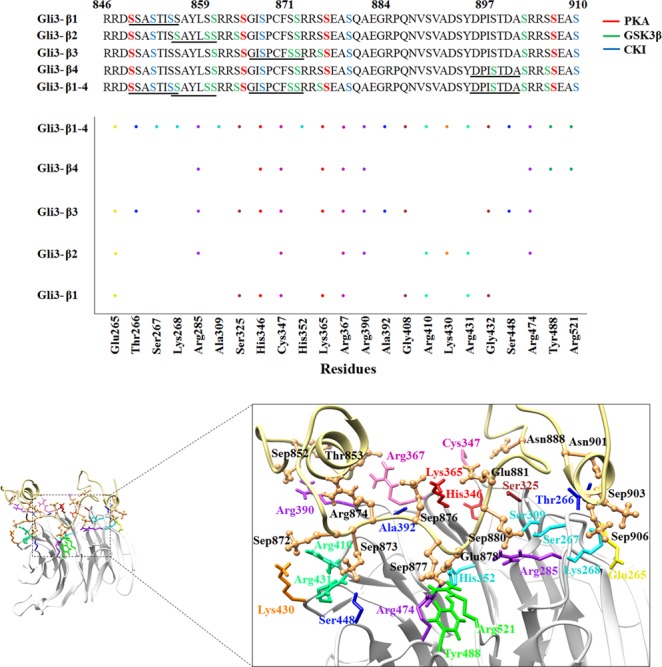
Structural details of βTrCP1 and GLI3 phosphopeptide binding. βTrCP1 is represented by light gray ribbon, while pale yellow ribbons represent phosphopeptide GLI3-β1–4 with interacting residues indicated by coral ball and stick mode. Illustration of four sequence motifs (β1 to β4) related to the βTrCP1 binding site are underlined that are phosphorylated by a putative cascade of PKA, GSK3β and CK1. PKA phosphorylated serines (phosphoserine) in the sequence motifs are colored in red. GSK3β phosphorylates serines (green) four residues N-terminal to a phosphoserine, while CK1 phosphorylates serines (blue) three residues C-terminal to a phosphoserine; both can chronologically multiphosphorylate GLI3 after priming. Middle panel shows the conservation pattern of βTrCP1 binding residues upon phosphopeptide binding. X-axis indicates the binding residues of βTrCP1 and Y-axis indicates the GLI3 phosphopeptides (GLI3-β1, GLI3-β2, GLI3-β3, GLI3-β4 and GLI3-β1–4). Dot represents the contribution of respective residue in binding to phosphopeptide.

Through comparative analysis of βTrCP1-bound phosphopeptides, contributions of βTrCP1-specific Ser267, Lys268, Ala309 and His352 residues were observed in GLI3-β1–4 phosphopeptide binding (Fig. 6). To further characterize the βTrCP1 and GLI3 phosphopeptide interactions, we mapped βTrCP1-specific probable regions that could be prerequisite for GLI3 phosphopeptide binding. Evidently, two residues (Cys347 and Arg367) lying in 3^rd^ WD40 repeat of βTrCP1 actively contributed in the phosphopeptide binding (Fig. 6). Additionally, Glu265, Arg285, Ser309, Ser325, Arg367, Arg390, Arg410, Lys430, Arg431, Ser448, Tyr488, Arg474 and Arg521 residues were directly involved in hydrogen bonding with phosphoserines of GLI3-β1–4.

**Figure 7:**
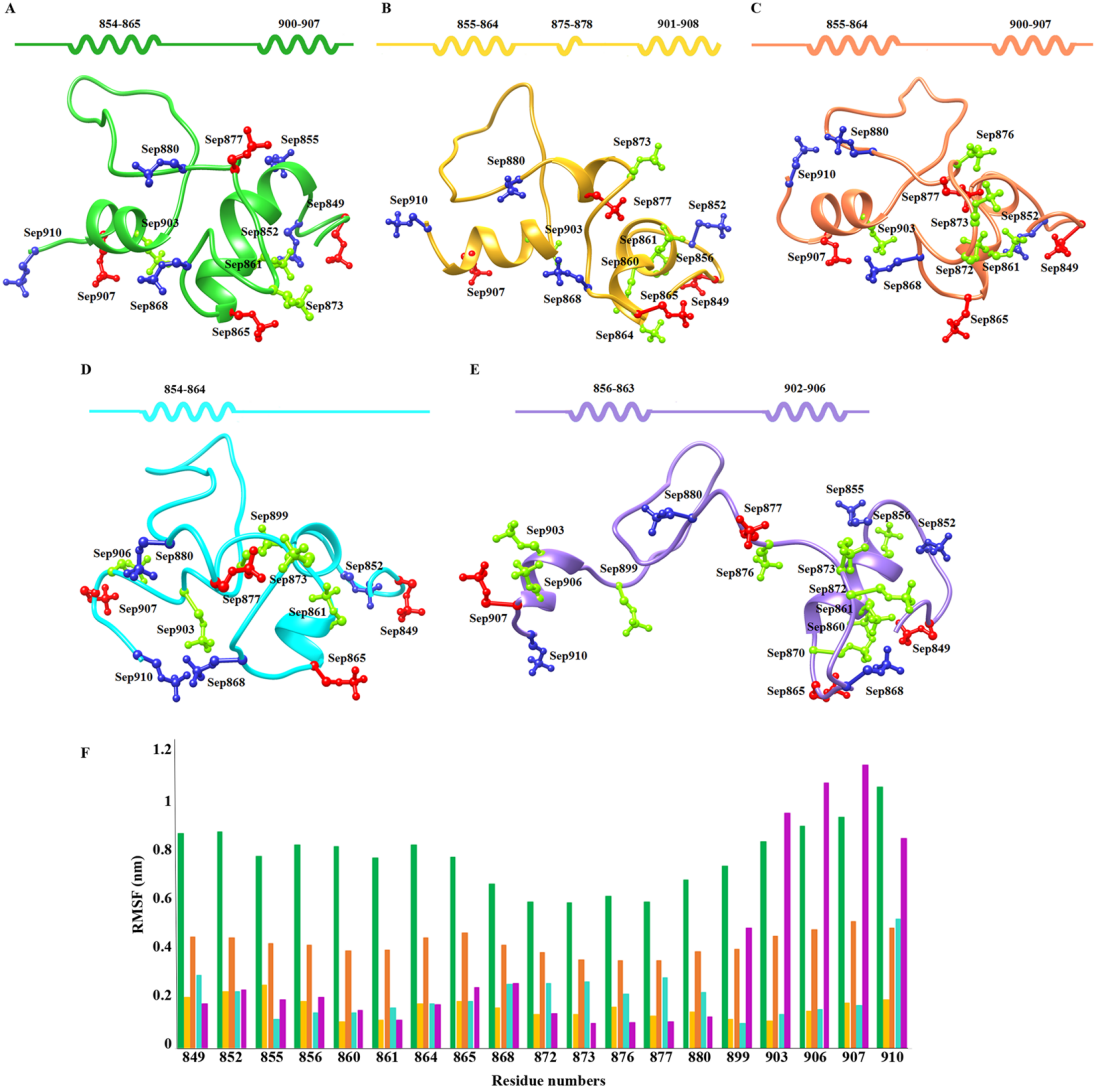
Conformational switches of the GLI3 phosphopeptide structure upon binding to βTrCP1. Phosphopeptides of (**A**) GLI3-β1, (**B**) GLI3-β2, (**C**) GLI3-β3, (**D**) GLI3-β4 and (**E**) GLI3-β1–4 are represented in green, gold, orange, cyan and purple colors, respectively. Phosphorylated residues via PKA, GSK3β and CSKI are shown by red, light green and blue colors, respectively in ball and stick mode. Secondary structures are illustrated above the corresponding plots. Coils delineate α-helices, while line specifies loop. (**F**) Comparative RMSF versus time plot of significant phosphorylated residues.

Furthermore, PDB files were characterized to measure the conformational switches in GLI3 phosphopeptides upon binding to βTrCP1. All phosphopeptides exhibited quite stable binding patterns at 25 ns. Particularly, upon binding to βTrCP1, both helical regions (Ile854-Ser864 and Thr900-Glu908) were shortened in GLI3-β1–4 to accommodate it in the cavity formed by β-propellers (Fig. 7E). A profound conformational change was observed in Thr900-Glu908 helical region (Fig. 7D), as upon binding to βTrCP1, this helix was completely missing. This trend was observed throughout MD simulation run as evident from the analysis of time-dependent secondary structure fluctuations via DSSP module (Fig. S3). Another notable secondary structural amendment was witnessed in the loop region of GLI3, where Sep875-Glu878 region of GLI3-β2 adopted a α-helical conformation upon binding to βTrCP1 (Figs S3B and 7B). In βTrCP1-bound GLI3-β1, GLI3-β3, GLI3-β4 and GLI3-β1–4 peptides, this region remained structurally preserved (Fig. S3). Subsequent analysis of RMSF indicated residual flexibility of phosphorylated residues upon GLI3 binding to βTrCP1. In case of GLI3-β1 and GLI3-β3 binding, major fluctuations up to 10 Å and 4.5 Å were perceived in all phosphorylated residues (Fig. 7F). Correspondingly, GLI3-β2 and GLI3-β4 peptides exhibited minor rate (up to 2.8 Å) of fluctuations as compared to other simulated systems. In case of GLI3-β1–4, significant fluctuations were detected in Sep899, Sep903, Sep906, Sep907 and Sep910 residues (4–11 Å) to assist in binding, while phosphorylated residues involved in binding (Sep852, Sep855, Sep872, Sep873, Sep876, Sep877 and Sep880) were quite stable (Fig. 7E). These results specified that Sep899-Sep910 of GLI3-β1–4 exhibited more fluctuations thus suggesting that Sep899-Sep910 region of GLI3 may be crucial for βTrCP1 binding.

### Binding free energy analysis

**Figure 8:**
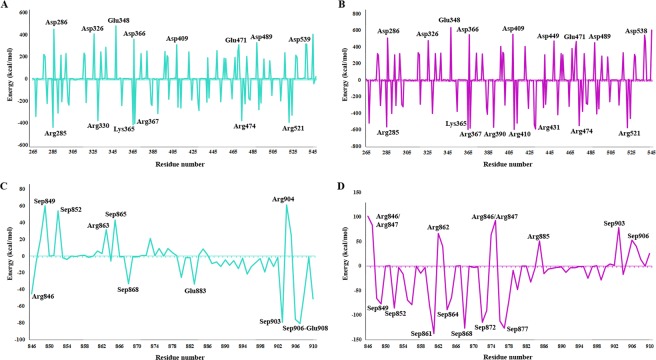
The binding free decomposition on per residue basis calculated from 40 ns MD trajectories by MM/PBSA method. Binding free energy decomposition at residue basis for βTrCP1 upon binding to (**A**) GLI3-β4 (**B**) GLI3-β1–4 peptides. Binding free energy decomposition on a per-residue basis for (**C**) GLI3-β4 (**D**) GLI3-β1–4.

βTrCP1 complexes with GLI3-β4 and GLI3-β1–4 were employed to estimate binding free energy values using MM/PBSA method. GLI3-β1–4 peptide possessed more negative binding free energy as compared to GLI3-β4, suggesting higher binding affinity for βTrCP1 (Table 4). The van der Waals (*E*_*vdw*_), electrostatic (*E*_*elec*_) interactions and nonpolar salvation (*ΔG*_*sol-nonpolar*_) energies negatively contributed, while polar solvation energy (*ΔG*_*sol-polar*_) contributed positively to the total binding energy (*ΔG*_*binding*_). Our results demonstrated a dominant role of electrostatic interaction in stabilizing the βTrCP1 and GLI3-β1–4 association. The binding free energy decomposition analysis revealed multiple residual contributions (Fig. 8), which delineated a similar interaction pattern with βTrCP1. These data were consistent with the findings of RMSF analysis (Fig. 4C). In case of GLI3-β1–4 and βTrCP1 complex, predominant energy contributions were due to Arg285, Lys365, Arg367, Arg390, Arg410, Arg431, Arg474 and Arg521 residues (Fig. 8B). Notably, energetic contribution of key gatekeeper residues (Arg474 and Arg524) was significant in the overall interaction paradigm, as describe previously^19^. Sep849, Sep852, Sep868, Sep872 and Sep877 residues of GLI3-β1–4 were critical for βTrCP1 binding; however, active role of these residues was not observed in the binding of GLI3-β4 and βTrCP1 (Fig. 8D).

**Table 4:** Free energy (kJ/mol) calculation for βTrCP1 in complex with GLI3 phosphopeptides.

Phosphopeptides	E_elec_	E_vdw_	G_sol-polar_	G_sol-non-polar_	∆G_binding_
GLI3-β4	−1324.607 +/− 187.963	−349.724 +/− 33.707	1464.516 +/− 144.885	−41.420 +/− 4.542	−251.235 +/− 157.966
GLI3-β1–4	−5393.633 +/− 313.253	−445.585 +/− 37.789	4141.518 +/− 226.246	−65.095 +/− 4.126	−1762.795 +/− 236.720

## Discussion

Ubiquitination plays a crucial role in Hh signaling activity via GLI proteins^2^ that act as zinc finger transcriptional effectors and regulate vertebrate development^18^. In the absence of Hh, phosphorylation-mediated ubiquitination keeps GLI3 in the repressor form. Despite the fact that mechanistic role of phosphorylation-mediated GLI3 degradation is well-established, the structural-functional paradigm is largely unknown. Here, we explored the potential role of multisite phosphorylation in the selective binding of βTrCP1 and GLI3 phosphopeptides via *in silico* approaches. Evidently, key substrate binding residues (Arg285, Arg307, Ser327, Ile344, Cys347, Lys365, Arg367, Asn394, Ser448, Arg474 and Arg521) of βTrCP1 were consistent with the earlier studies^19,42^. RMSD analysis demonstrated stability (2–4 Å) in all systems at 12 ns. Further analysis elucidated multiple conformational changes that invoked specificities in the β-propeller upon phosphopeptide binding. The overall topology of β-strands remained preserved in the βTrCP1 structure (Table 3). A predominant transformation of β-strand (Thr381-Leu386) into loop conformation facilitated the binding via flexibility. Other prominent positional readjustments observed in the β-strands were localized in WD40 repeat-1 (Arg301-Leu303 and Leu313-Tyr315) and repeat-6 (Ile492-Trp495 and Ile532-Ser534), leading to GLI3-β1–4 binding. In RMSF analysis, βTrCP1 binding region (Arg285-Arg521) attained more stability upon binding to GLI3-β-4 (Fig. 4D).

In agreement to the previous observations^17^, where crucial role of GLI3 motif-4 has been reported in βTrCP1 binding and GLI3 processing, our findings indicate that motif-4-specific Ser899 phosphorylation invokes other phosphorylated serines to impart active role in binding to βTrCP1 (Fig. 8). The interaction of βTrCP1 and GLI3 was significantly influenced by the positional readjustments of residues lying in two helices (Ile854-Sep865 and Thr900-Sep907) due to phosphorylation of paired neighboring residues that induced flexibility differences through helix-loop inter-conversion. Generally, introduction of phosphate group targets loop conformation by rearranging the hydrogen bonding network of side chains lying at the vicinity of loop region^48^. These transitions in the surrounding regions render helical shifting into loop that acts as a conformational switch for the binding cleft geometry^49^. The presence of diverse hydrogen bonding pattern and conformational switching due to phosphorylation is crucial for the recognition of GLI3 by βTrCP1. Any change in this pattern may impair their binding affinity due to imbalanced phosphorylation level. It is however unclear at the moment how energy barrier overcomes the phosphorylation or other post-translational modification-induced conformational space.

GLI3 contains multiple binding sites for βTrCP1, where approximately, two-third of GLI3 contacts involve phosphorylated PKA sites and secondary CK1/GSK3 sites. The potential involvements of GLI3-specific primary (Ser852, Ser873, Ser877, Ser880 and Ser903) and secondary phosphorylated (Ser855, Ser872, Ser876, Ser906) residues in βTrCP1 binding indicate that both primary and secondary phosphorylations are required for βTrCP1 binding. Study of interdependent phosphorylation status through structural knowledge may expand the repertoire of GLI3 processing. Indeed, any mutation at the PKA-specific sites may significantly reduce the binding of βTrCP1 to GLI3^7^. Taken together, our results are in good agreement with the experimental outcomes^7,17^. This study may uncover the spectrum of structural linkages in association with the kinase-mediated phosphorylation paradigm to illustrate the molecular basis of GLI3 processing in Hh signaling. Further studies will be needed to elaborate the effect of putative phosphorylation site mutations at structural level.

## Supplementary information

supplementary data

## Data Availability

All data generated or analyzed during this study are included in this published article (and its supplementary information files).
