# Supplementary material for: Structural basis of βTrCP1-associated GLI3 processing
Source: Sci Rep. 2019 May 3;9:6865. doi: 10.1038/s41598-019-43392-3 (PMC6499770; doi:10.1038/s41598-019-43392-3)
Supplement: Supplementary file 1 — supplementary data [file 41598_2019_43392_MOESM1_ESM.docx]

**Title: Structural basis of βTrCP1-associated GLI3 processing**

Authors: Shagufta Shafique, Sajid Rashid*

**Supplementary data**

**
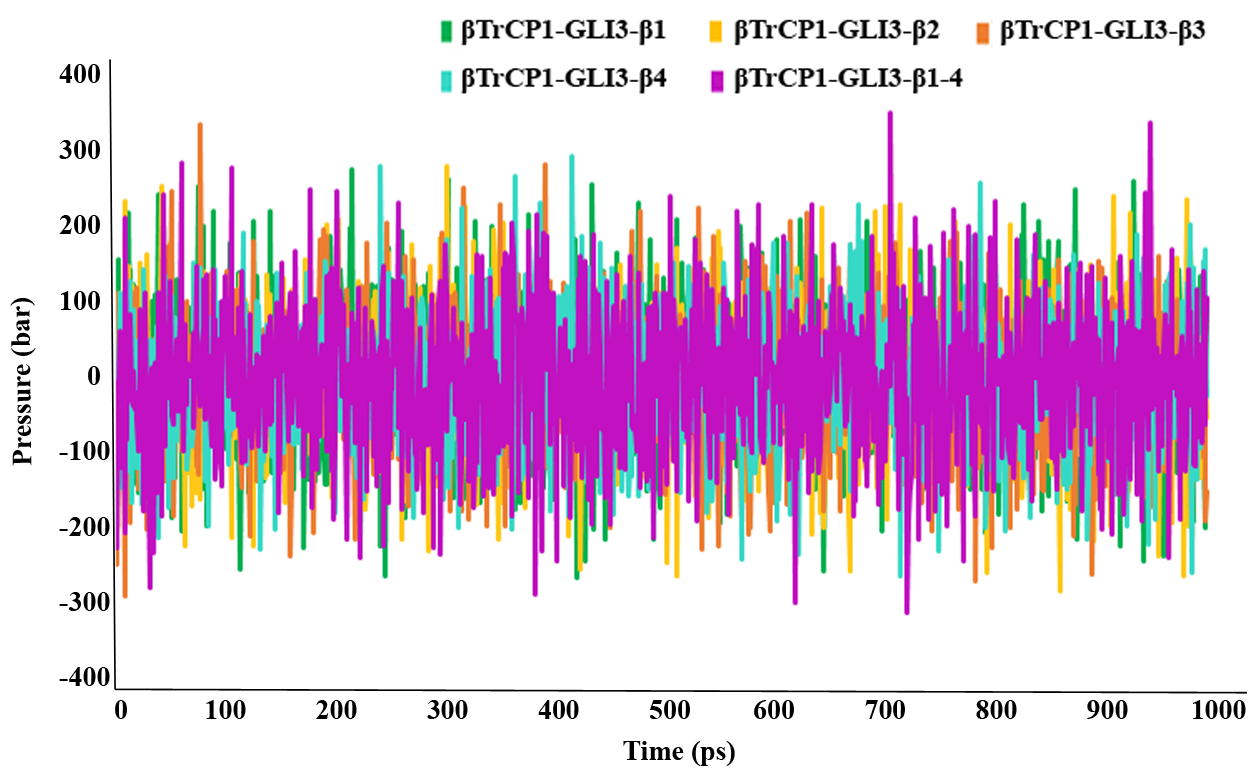
**

**Figure S1. Time-dependent analysis of equilibrated systems during MD simulations of GLI3 peptide-bound βTrCP1.**


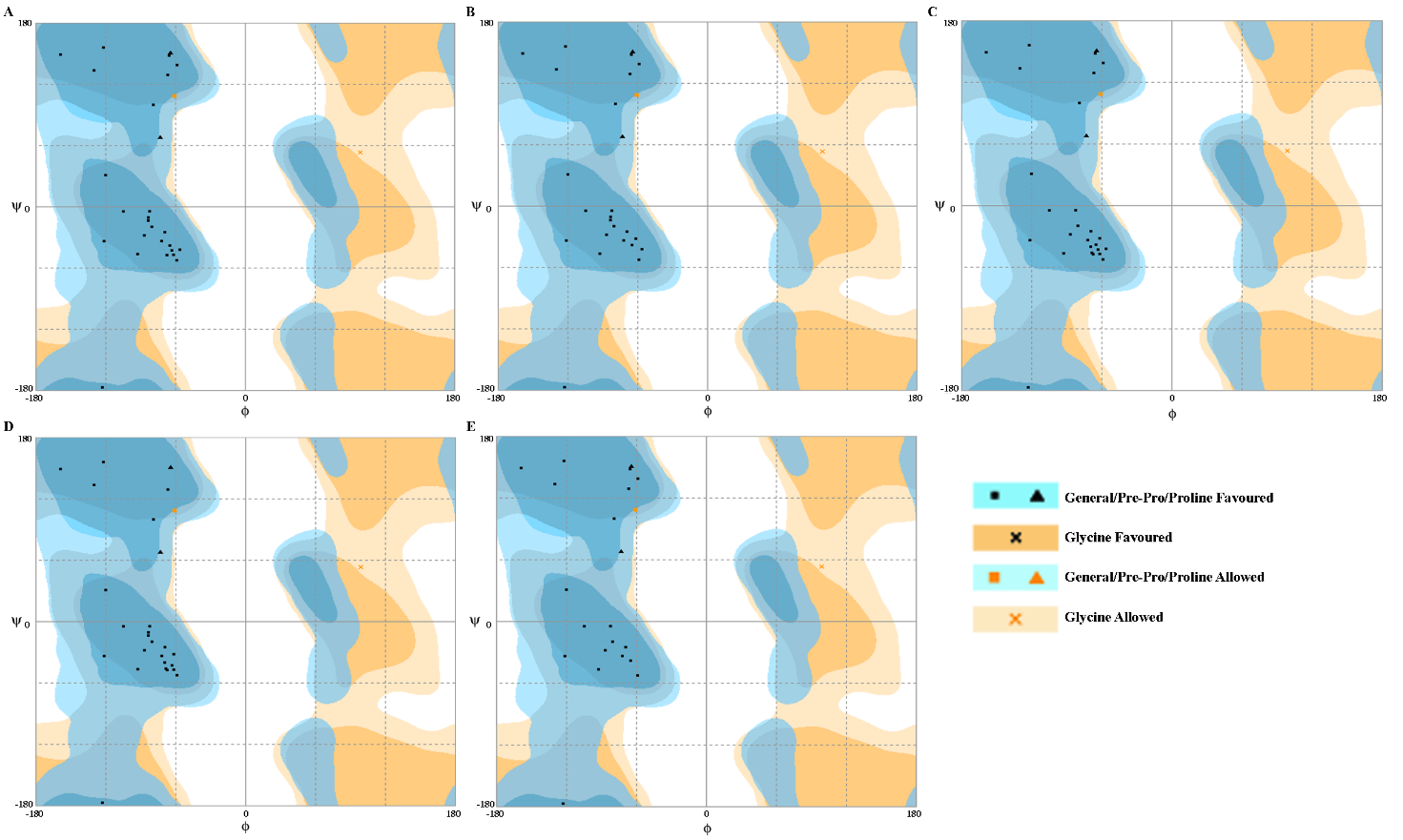
**Figure S2. Ramachandran plot of GLI3 phosphopeptides (A) GLI3-β1, (B) GLI3-β2, (C) GLI3-β3, (D) GLI3-β4 and (E) GLI3-β1-4.**

**
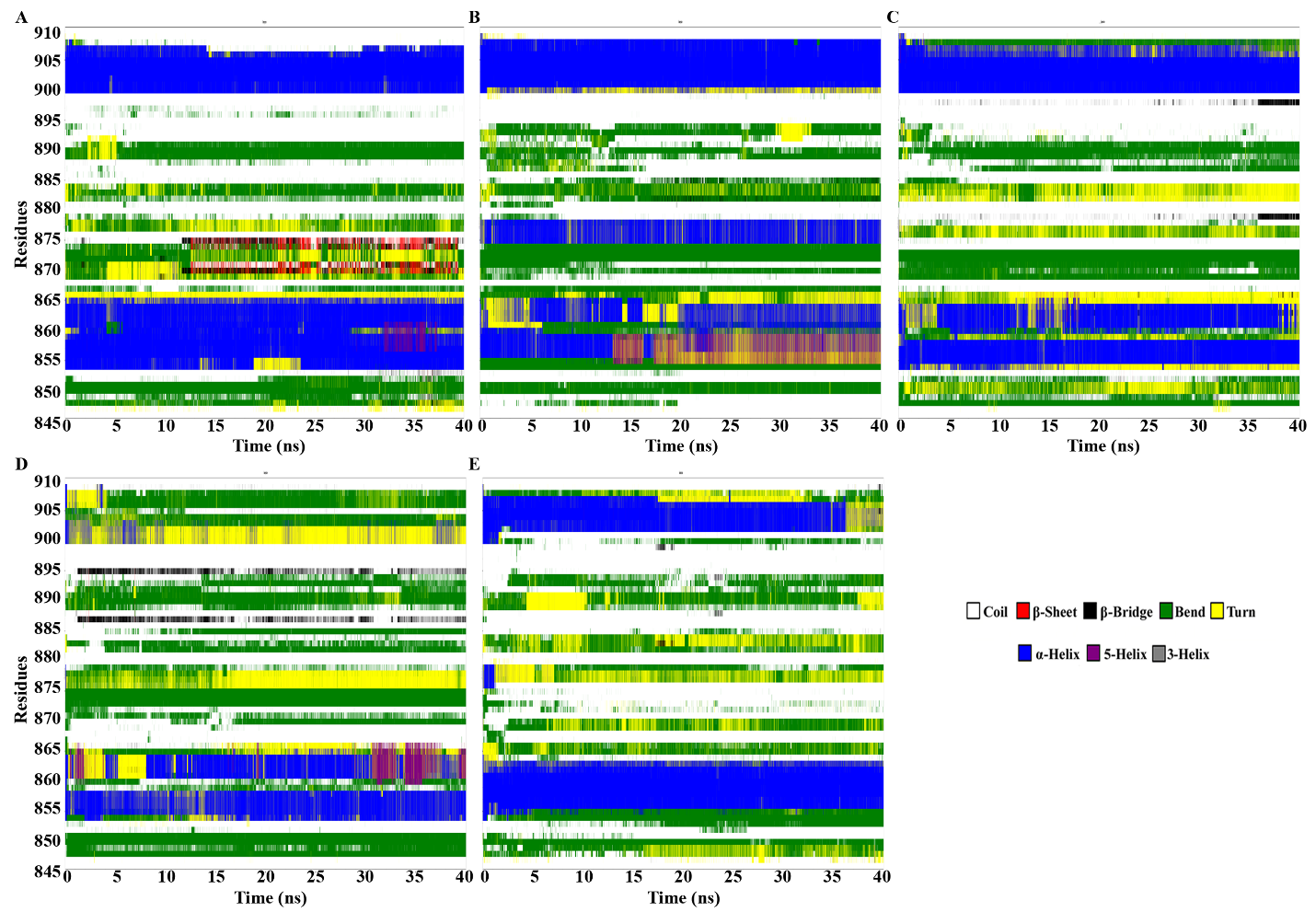
Figure S3. DSSP specific secondary structure assignment for 40 ns total simulation time. (A) GLI3-β1, (B) GLI3-β2, (C) GLI3-β3, (D) GLI3-β4 and (E) GLI3-β1-4.**
